# The initiator of neuroexcitotoxicity and ferroptosis in ischemic stroke: Glutamate accumulation

**DOI:** 10.3389/fnmol.2023.1113081

**Published:** 2023-03-23

**Authors:** Genhao Fan, Menglin Liu, Jia Liu, Yuhong Huang

**Affiliations:** ^1^Graduate School, Tianjin University of Chinese Medicine, Tianjin, China; ^2^Department of Clinical Pharmacology, The Second Affiliated Hospital of Tianjin University of Chinese Medicine, Tianjin, China

**Keywords:** neuroexcitotoxicity, ferroptosis, glutamate, cystine-glutamate antiporter, ischemic stroke

## Abstract

Glutamate plays an important role in excitotoxicity and ferroptosis. Excitotoxicity occurs through over-stimulation of glutamate receptors, specifically NMDAR, while in the non-receptor-mediated pathway, high glutamate concentrations reduce cystine uptake by inhibiting the System Xc-, leading to intracellular glutathione depletion and resulting in ROS accumulation, which contributes to increased lipid peroxidation, mitochondrial damage, and ultimately ferroptosis. Oxidative stress appears to crosstalk between excitotoxicity and ferroptosis, and it is essential to maintain glutamate homeostasis and inhibit oxidative stress responses *in vivo*. As researchers work to develop natural compounds to further investigate the complex mechanisms and regulatory functions of ferroptosis and excitotoxicity, new avenues will be available for the effective treatment of ischaemic stroke. Therefore, this paper provides a review of the molecular mechanisms and treatment of glutamate-mediated excitotoxicity and ferroptosis.

## 1. Introduction

Ischemic stroke is the primary cause of death and disability in Chinese adults, characterized by high morbidity, disability, mortality, and recurrence rate (Sturm et al., 2002; Gao J. et al., 2022). According to statistics, the age-standardized prevalence of stroke in China in 2013 was 1114.8 per 100,000, with an incidence rate of 246.8 per 100,000 and a mortality rate of 114.8 per 100,000 (Wang W. et al., 2017), The Continuous Stroke Surveillance Program in 31 Chinese provinces reported an annual increase of 8.3% in the incidence of first stroke in adults, from 189 cases per 100,000 people in 2002 to 379 cases per 100,000 people in 2013，with the incidence of ischaemic stroke and hemorrhagic stroke at 335 per 100,000 population and 44 per 100,000 population, respectively, in 2013 (He et al., 2022). In the United States, more than 795,000 people suffer a stroke each year, accounting for about one in 10 deaths in the United States, and is the leading cause of long-term disability in the country (Engler-Chiurazzi et al., 2017; Ho et al., 2019; Barthels and Das, 2020). By 2050, more than 150 million people worldwide will be 65 and over (Feigin et al., 2014; Thomazi et al., 2018; He and Zhou, 2020)，the number of people suffering from stroke is expected to increase steadily in the coming decades as the population ages (Boudreau et al., 2013; Ji et al., 2022; Kevdzija et al., 2022; Tsao et al., 2022; Zhou et al., 2022).

**Figure 1:**
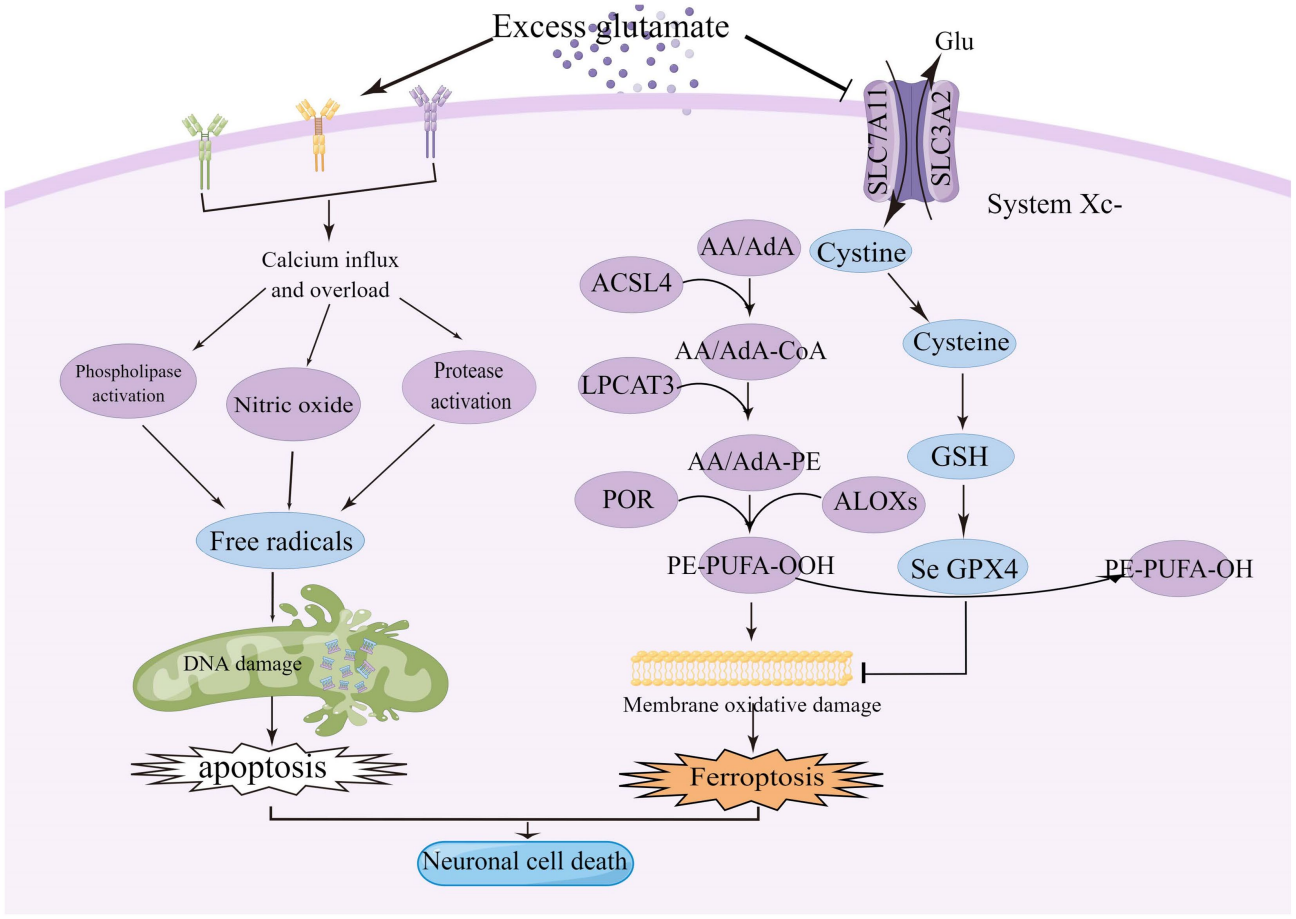
Excitotoxicity is caused by over-stimulation of glutamate receptors, particularly NMDAR, leading to high calcium influx, mitochondrial dysfunction, and DNA breakage. High levels of glutamate reduce the uptake of cystine *via* the Xc-system, leading to intracellular glutathione depletion resulting in the accumulation of reactive oxygen species (ROS), which increases lipid peroxidation, mitochondrial damage and ultimately ferroptosis. ACSL4, acyl-coenzyme A synthase long chain family member 4; System Xc-, cystine/glutamate reverse transporter; LPCAT3, lysophosphatidylcholine acyltransferase 3; AA, arachidonic acid; AdA, adrenoyl acid; ALOXs, lipoxygenases; CoA, coenzyme A; POR, cytochrome p450 oxidoreductase; GPX4, glutathione peroxidase 4; and GSH, glutathione.

The central premise of ischaemic stroke treatment is to limit infarction by rapid and effective recanalization of occluded vessels, leading to reperfusion of the ischaemic semidark zone, and there have been significant advances in the treatment of patients with ischaemic stroke over the last decade or so of research (Bivard et al., 2017; Malysz-Cymborska et al., 2021). Currently, drugs commonly used to treat ischaemic stroke include drugs to improve cerebral circulation, neuroprotective agents, and herbs to activate blood circulation and resolve blood stasis. The only thrombolytic medication that has received FDA approval is tissue fibrinogen activator (tPA), but its clinical application is restricted to a certain time window (Fukuta et al., 2017; Hu et al., 2022; Yoon et al., 2022). A recent meta-analysis of individual participant data on alteplase showed that, regardless of age or stroke severity, giving alteplase within 4–5 h of stroke onset significantly improved the overall odds of a good stroke prognosis, despite an increased risk of fatal intracranial hemorrhage within a few days of treatment, and the earlier the treatment, the greater the proportion of benefit. However, recanalisation success rates were lower with intravenous administration of alteplase, thus reducing overall efficacy (Emberson et al., 2014; Leiva-Salinas et al., 2016). Although numerous studies have shown that inflammation, oxidative stress, excitotoxicity, calcium overload, apoptosis, and disruption of the blood–brain barrier are causative mechanisms of ischaemic stroke, preclinical protective agents targeting one of these mechanisms have not been used in the clinic (Dirnagl et al., 1999; He et al., 2013). Therefore, there is an urgent need to better understand the physio-pathological mechanisms that regulate these complex molecular effects in order to facilitate the research and development of new drugs and improve patient prognosis (Zou et al., 2022). This article critically discusses the role of glutamate receptor-mediated excitotoxicity and cystine/glutamate antiporter-mediated ferroptosis in ischemic stroke, as shown in Figure 1.

## 2. Glutamate receptor-mediated excitotoxicity

### 2.1. The role of glutamate in synaptic transmission

Glutamate is the main excitatory neurotransmitter in the central nervous system (CNS) and is closely linked to synaptic activity, plasticity, cell death and survival, learning and memory, and pain perception (Byrnes et al., 2009; Arteaga Cabeza et al., 2021). Excitotoxicity, a toxic effect of excessive or prolonged glutamate activation of the receptor, was first studied by Dr. Olney (Wang S. et al., 2017; McCaughey-Chapman and Connor, 2022). Excitotoxicity, excessive and pathological stimulation of neurons, associated with neuronal death in many neurological diseases, including ischaemia, traumatic brain injury, and neurodegenerative diseases (Connolly et al., 2016; Krasil'nikova et al., 2019). All intercellular signaling is dependent on chemical signals, and glutamate is one of the most important intercellular chemical signals in the nervous system (Özel et al., 2014; Teng et al., 2016).

**Figure 2:**
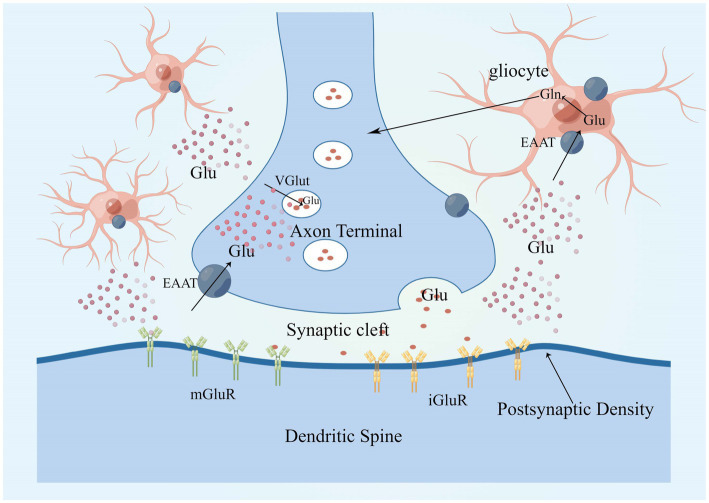
Glutamate in neurons is concentrated in synaptic vesicles *via* VGLUT and is released into the extracellular space in response to neuronal depolarization. There are two main classes of glutamate receptors, that is, mGluRs and iGluRs, glutamate clearance from the extracellular space takes place mostly through the high-affinity EAATs, EAAT 1 and 2 are mainly expressed in astrocytes. Glutamate enters glial cells *via* EAAT1 and EAAT2, where it is metabolized to glutamine, which is released into the extracellular space and converted to glutamate after uptake by neurons, completing a cycle. EAAT, excitatory amino acid transporter; mGluR, metabotropic glutamate receptor; iGluRs, ionotropic glutamate receptors; VGLUT, vesicular glutamate transporters; and PSD, postsynaptic density.

Glutamate is approximately 5–15 mmol/kg in brain tissue, 5–10 mM in neurons, and 30–50 μM in plasma, with glutamate concentrations fluctuating in response to body metabolism, diet, etc. (Bramham et al., 1990; Ottersen et al., 1990, 1992; Osen et al., 1995; Danbolt, 2001). Glutamate concentrations in neurons are highest at axon terminals, which means that axon terminals somehow restrict glutamate movement or local synthesis and utilization of glutamate, and glutaminase is responsible for glutamate synthesis in most neurons (Márquez et al., 2009; Barbano et al., 2020; Pietrancosta et al., 2020). Glutamate in neurons is concentrated in synaptic vesicles *via* the vesicular glutamate transporter (VGLUT) and released into the extracellular space when the neuron is depolarized (Takamori et al., 2000; Takamori, 2006). Glutamate concentrations are highest in teleneuron and up to 100 mM in synaptic vesicles (Riveros et al., 1986; Burger et al., 1989; Shupliakov et al., 1992). When an action potential reaches the presynaptic terminal, Ca^2+^ influx *via* voltage-gated calcium channels (VGCC) triggers the fusion of vesicles loaded with neurotransmitter with the cell membrane, thereby releasing neurotransmitter in the synaptic cleft (Nishimune et al., 2016; Liang et al., 2021; Tukker and Westerink, 2021; Fedorovich and Waseem, 2022). Glutamate is secreted into the synaptic gap where it can diffuse around the neuron and interact with surrounding targets (Clewett et al., 2017), closest to the axon terminal is the postsynaptic membrane, which contains a large number of membrane-associated proteins, these “postsynaptic densities (PSD)” can be seen under the electron microscope (Kennedy, 1997; Xu Y. et al., 2021), PSDs contains a large number of glutamate receptors, which bind to glutamate and then trigger the postsynaptic cell to complete the synaptic transmission of glutamate signals from the presynaptic to the postsynaptic cell (Guo and Cordeiro, 2008; Terauchi and Umemori, 2012; Katayama et al., 2017). The transport pattern of glutamate is shown in Figure 2.

### 2.2. Type of glutamate receptor and mechanism of action

Excitotoxicity was one of the first mechanisms of ischemic cell death to be identified and one of the most intensively studied, with the term “excitotoxicity” describing the process by which excess glutamate overactivates NMDA receptors (NMDARs) and induces neuronal toxicity (Choi et al., 1988; Garthwaite et al., 1992). There are two types of glutamate receptors: ionotropic glutamate receptors (iGluRs), which are ligand-gated ion channels, and metabotropic glutamate receptors (mGluRs), which are G protein-coupled receptors (Pin and Duvoisin, 1995; Ferraguti and Shigemoto, 2006). The ionotropic receptors include kainate (KA) receptors, alpha-amino-3-hydroxy-5-methyl-4-isoxazole propionic acid (AMPA) receptors, and N-methyl-D-aspartate (NMDA) receptors (Takahashi, 2019; Burada et al., 2020). iGluRs are ligand-gated ion channels that allow cations such as calcium and potassium to cross the plasma membrane after glutamate binding to the receptor (Wei et al., 2011; Rocha-Ferreira and Hristova, 2016).

**Figure 3:**
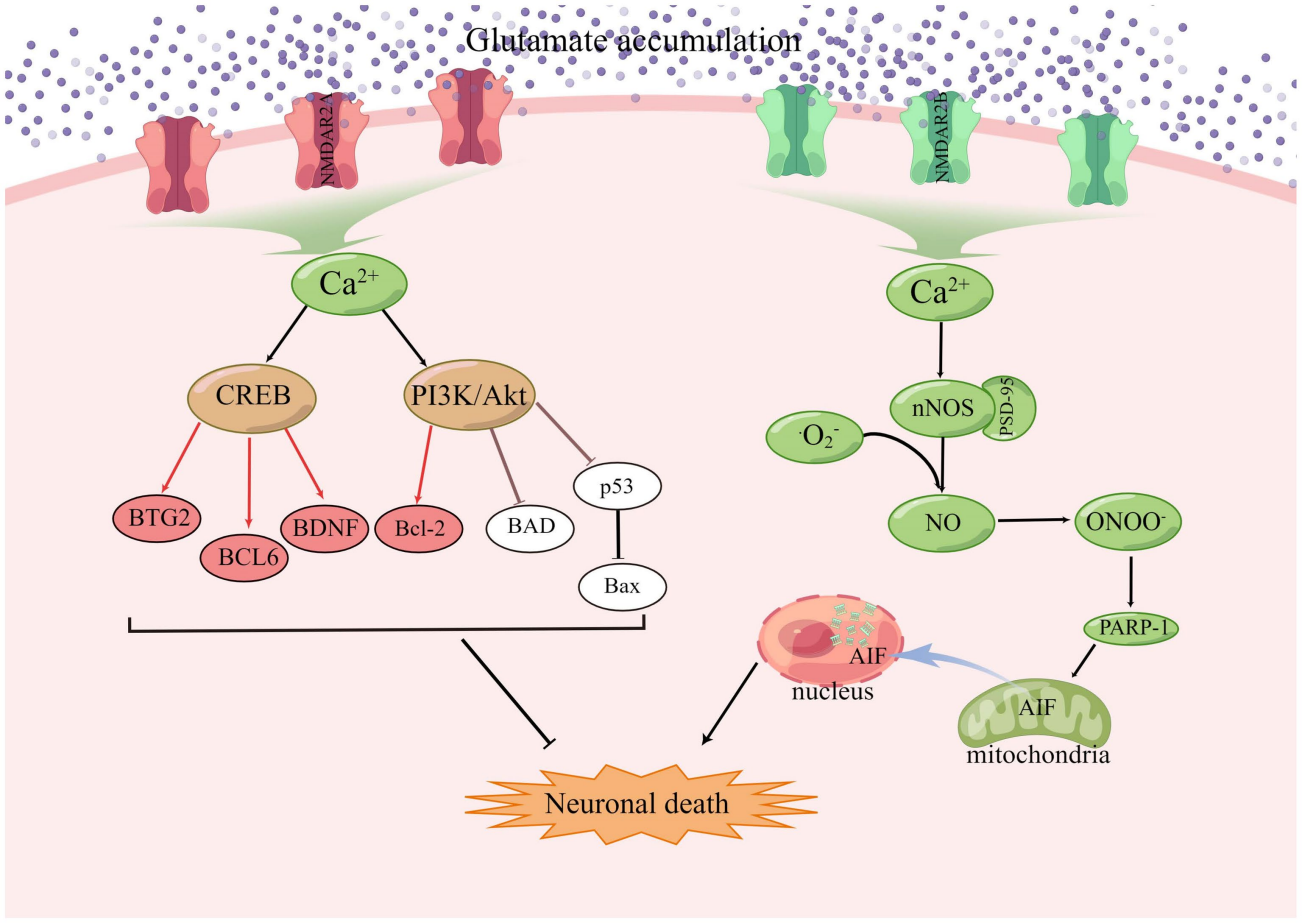
In ischaemic stroke, GluN2A and GluN2B play opposing roles, with GluN2B activation leading to excitotoxicity and apoptosis and GluN2A activation promoting cell survival.

NMDA receptors require a basic NR1 subunit and one or more regulatory NR2 subunits (NR2A-D), and also NR3 subunits (NR3A-B), in some specific cases (Ye et al., 2013; Rebas et al., 2020). In the resting state, NMDAR channels are normally blocked by Mg^2+^, but when large amounts of glutamate accumulate, activated AMPAR causes partial depolarization of the postsynaptic membrane, sufficient to clear the Mg^2+^ on the NMDAR. Among the currently known ionotropic and metabotropic glutamate receptors, NMDAR play an important role in allowing excess Ca^2+^ inward flow, leading to ischemic cell death (Mao et al., 2022). Calcium overload activates a large number of downstream pro-death signals such as calpain activation, reactive oxygen species (ROS) production, and mitochondrial damage (Fujimura et al., 1998; Kristián and Siesjö, 1998; Eliasson et al., 1999; Lau and Tymianski, 2010), resulting in cell necrosis or apoptosis (Köhr, 2006; Shi et al., 2017; Yoo et al., 2017; Maher et al., 2018). GluN2A and GluN2B play opposite roles in ischaemic stroke, with activation of GluN2B leading to excitotoxicity and neuronal apoptosis, while activation of GluN2A protects neurons (Liu et al., 2007; Chen et al., 2008). Under stress conditions, NMDAR2A activates the PI3K/Akt kinase pathway, promoting the expression of cAMP response element binding protein (CREB) related genes and inhibiting the expression of pro-death genes, and Akt promotes cell survival by phosphorylating many downstream targets (Wu and Tymianski, 2018). Akt also inactivates the pro-apoptotic Bcl-2 family member BAD (Bcl2/Bcl-XL-antagonist causing cell death) by phosphorylation, thus stopping its interaction with and blockade of the pro-survival Bcl-2 family members Bcl-2 and Bcl-XL (Papadia and Hardingham, 2007). The JNK/p38 activator ASK1 is also inhibited by phosphorylation by Akt, and the activity of p53 is inhibited by Akt, resulting in reduced Bax expression (Kim et al., 2001; Yamaguchi et al., 2001). CREB target genes include the anti-apoptotic BTG2, the apoptotic p53 inhibitor BCL6, and the neurotrophic factor BDNF (Hardingham et al., 2002; Hardingham, 2009). During synaptic contact, these receptors are present in high density in a specific region of the postsynaptic membrane, which is closely associated with the presynaptic active zone of glutamate release (Sheng and Hoogenraad, 2007). PSD-95 was found to bind to NMDAR2B and intracellular neuronal nitric oxide synthase (nNOS) as part of a scaffold synaptic protein, and in the presence of intracellular calcium, PSD-95 plays a crucial role in the mechanism by which NMDAR activity triggers the production of nitric oxide production by nNOS and excitotoxicity (Cui et al., 2007; Forder and Tymianski, 2009; Abergel, 2020). NO combines with superoxide radicals to produce large amounts of nitrite, which leads to protein oxidation, lipid peroxidation, and DNA damage (Lipton et al., 1993), as shown in the Figure 3.

AMPAR is constructed from four subunits (GluR1-4; Hwang and Lupica, 2020; Zhang et al., 2022). Under resting conditions, the NMDAR channel pores are blocked by Mg^2+^ ions and once sufficient membrane depolarization has been established, the Mg^2+^ block is removed, allowing the influx of cations (Lin et al., 2008; Olive, 2009).AMPAR activation increases Na^+^ influx into neurons, depolarizes membranes, and activates voltage-dependent Ca^2+^ channels and NMDARs (Andriessen et al., 2010), the substitution of a positively-charged arginine residue for a neutrally-charged glutamine residue at the apex of the membrane reentrant pore loop (M2) changes the conductance properties of channels containing an edited GluR2 subunit (Köhr et al., 1998; Hood and Emeson, 2012). Most GluR2 subunits expressed in the mature rat cochlea are edited form and therefore, when incorporated into AMPA receptors, render the GluR complex calcium impermeable (Carriedo et al., 1996; Graham et al., 2011; Basappa et al., 2012). Molecular cloning has identified five isoforms, named GluK1, GluK2, GluK3, GluK4, and GluK5 according to the new IUPHAR nomenclature, which form functional receptors in various combinations (Dingledine et al., 1999).

## 3. Excess glutamate accumulation can inhibit the cystine/glutamate reverse transporter and lead to ferroptosis

Since 2005, the Nomenclature Committee on Cell Death (NCCD) has updated the classification system and in 2018 introduced an updated version based on molecular mechanisms, in which cell death is divided into two parts, accidental cell death (ACD), and regulated cell death (RCD; Galluzzi et al., 2018; Čepelak et al., 2020). There are several types of RCD, including apoptotic and non-apoptotic (Shen et al., 2022). Ferroptosis cells often show a necrotic appearance, such as cell swelling, plasma membrane rupture, and mitochondrial damage, unlike apoptotic cells, which are characterized by membrane blistering and contraction (Hou et al., 2021). Ferroptosis is a newly identified form of cell death caused by iron-dependent lipid peroxidation. Which leads to cell membrane damage and the accumulation of reactive lipid hydroperoxides to lethal levels (Munro et al., 2022). Our original knowledge of the molecular mechanisms of ferroptosis stemmed from studies using small molecule compounds to selectively inhibit cancer cells with oncogenic RAS mutations (Chen et al., 2021; Andreani et al., 2022). Ca^2+^ plays a fundamental role in glutamate-mediated excitotoxicity or oxidation-mediated cell death, a form of programmed cell death similar to or possibly identical to ferroptosis (Tan et al., 2001; Maher et al., 2018). Inhibiting System Xc- and inactivating GSH peroxidase-4 (GPX4) causes cellular glutathione (GSH) depletion and impaired ROS scavenging, resulting in disruption of cellular redox homeostasis, accumulation of ROS in the lipid peroxidation or Fenton reaction, and ultimately cell death (Shi et al., 2021).

### 3.1. Characteristics of ferroptosis

Ferroptosis cells undergo morphological changes at both the cellular and ultrastructural levels: the plasma membrane loses its integrity, the cytoplasm becomes enlarged, the mitochondria become smaller than normal cells, the mitochondrial cristae shrink or disappear, the outer mitochondrial membrane ruptures and the membrane density increases (Dolma et al., 2003; Yagoda et al., 2007; Dixon et al., 2012; Friedmann Angeli et al., 2014; Vanden Berghe et al., 2014). Mitochondria are an important source of ROS. Recent studies have found that impaired mitochondrial function leading to ROS production, DNA stress, and metabolic reprogramming is responsible for lipid peroxidation and ferroptosis (Gao et al., 2019; Lee et al., 2020; Li et al., 2021). Ferroptosis is mainly associated with iron accumulation and lipid peroxidation. Excess iron combines with hydrogen peroxide in a Fenton reaction to produce large amounts of hydroxyl radicals, increasing oxidative damage. Iron also increases the activity of lipoxygenase (ALOX) or prolyl hydroxylase (PHD), further aggravating lipid peroxidation (Chen X. et al., 2020; Lin et al., 2021; Tang D. et al., 2021). Lipid peroxidation occurs as a free radical-driven reaction that primarily affects the metabolism of polyunsaturated fatty acids (PUFAs) in cell membranes (Gao Q. et al., 2022; Nie et al., 2022). Lipopolymer peroxidation products include the initial lipid hydroperoxide (LOOH) and the subsequent reactive aldehyde (MDA,4-HNE), which increase during ferroptosis (Nie et al., 2022). The PTGS2 gene encodes prostaglandin endoperoxide synthase (PTGS), a key enzyme in prostaglandin biosynthesis (Yang et al., 2014). Acyl-Coenzyme A synthase long chain family member 4 (ACSL4) is thought to be a specific biomarker and driver of ferroptosis as it is a key enzyme involved in fatty acid metabolism. Upregulation of ACSL4 leads to an increase in polyunsaturated fatty acid content in phospholipids, which are particularly susceptible to oxidative reactions and ultimately ferroptosis (Yuan et al., 2016; Doll et al., 2017). Activation of transcriptional pathways of genes responsible for antioxidant defense (GSH, CoQ10, and NRF2) and membrane repair (ESCRT-III) limits membrane damage during ferroptosis (Dixon et al., 2012; Sun et al., 2016; Bersuker et al., 2019; Doll et al., 2019; Dai et al., 2020). The dynamic balance between damage and resistance to damage determines the survival or death of cells.

### 3.2. Critical role of amino acid metabolism and lipid metabolism in ferroptosis

#### 3.2.1. Amino acid metabolism

Cystine/glutamate reverse transporter (System Xc-) is an amino acid reverse transporter protein that mediates the inward flow of cystine and the outward flow of glutamate (Kagami et al., 2018; Wang et al., 2020; Marcoli et al., 2022). The cystine taken into the cell is reduced to cysteine, part of which participates in intracellular GSH synthesis and the other part flows out of the cell to be converted to cystine and re-involved in the System Xc- (Liu N. et al., 2020; Tang Z. et al., 2021). Glutathione is an antioxidant and an important indicator of oxidative stress in cells (Guo et al., 2012). When there is too much extracellular glutamate, it inhibits the function of the System Xc-, resulting in less cystine entering the cell, which is an excitatory neurotransmitter with neurotoxic and excitatory effects (Liao et al., 2018; Ratan, 2020). System Xc- mediates the uptake of cystine and the release of glutamate, thereby promoting the synthesis of GSH, which acts as a co-molecule with GPX-4 to assist in the scavenging of lipid peroxides to protect cells (Zhao et al., 2021). System Xc- is a heterodimeric protein consisting of one light chain and one heavy chain with a disulfide bond between the two chains (Chen et al., 2022; Wang Y. et al., 2022). The light chain subunit SLC7A11 is the primary transporter and is highly sensitive to cystine and glutamate, while the heavy chain subunit SLC3A2 acts essentially as a chaperone protein and plays an important role in the transport of SLC7A11 to the plasma membrane (Koppula et al., 2018). SLC7A11 is a 12-channel transmembrane protein with both its N and C termini in intracellular locations, whereas SLC3A2 is a single-transmembrane protein with its N terminus in intracellular locations and its c terminus in extracellular locations (Sato et al., 1999; Xu C. et al., 2021; Chen et al., 2022). In addition, Knockdown of SLC3A2 has been shown to result in a significant lowering of SLC7A11 protein levels, suggesting that SLC3A2 is critical in sustaining SLC7A11 protein stability (Nakamura et al., 1999; Shin et al., 2017; Koppula et al., 2018). Intracellular cysteine is an essential precursor of glutathione. Glutathione is a tripeptide synthesized by cysteine, glutamate and glycine (Koppula et al., 2018; Gan, 2019; Zhao et al., 2022). The biosynthesis of GSH involves two crucial steps, first by formation of gamma-glutamylcysteinyl linkage by formation of gamma-glutamyl cysteine, followed by the addition of glycine *via* glutathione synthase (GSS) to produce the tripeptide glutathione (Raza et al., 2022). Endogenous enzymes protect cells from damage caused by excess ROS, including superoxide dismutase which converts superoxide (O_2_^−^) to hydrogen peroxide (H_2_O_2_), glutathione peroxidase (GPX) which converts free H_2_O_2_ to water, glutathione reductase which converts glutathione disulfide to the sulfhydryl form and catalytic breakdown of H_2_O_2_ to water and oxygen by peroxidase (Mahmoud et al., 2014). Oxidation of glutathione by the action of GPX and reduction of glutathione by glutathione reductase (GR) at the expense of NADPH (Koppula et al., 2018). Thus, System Xc- is critically important for the uptake of cystine to produce cysteine for the maintenance of intracellular GSH levels.

Two transcription factors were identified that regulate SLC7A11, nuclear factor red lineage 2-related factor 2 (NRF2) and activating transcription factor 4 (ATF4). NRF2 is a master transcription factor that accounts for antioxidant responses (Becker et al., 2016; Kuo et al., 2022). Under normal physiological conditions, Nrf2 is ubiquitinated by the Keap1-Cullin3 ubiquitin ligase complex and is conventionally fragmented by the 26 s proteasome. In contrast, under oxidative stress conditions, ubiquitin ligase activity is blocked by modifying the cysteine residues in Keap1, thereby stabilizing and activating (Noguchi et al., 2018), stable NRF2 then translocates into the nucleus, binds to antioxidant response elements in the gene promoter region and regulates the transcription of a range of target genes involved in antioxidant defense and cellular redox maintenance (Ooi et al., 2018; Koppula et al., 2021a), Similarly, overexpression of NRF2 upregulated the expression levels of antioxidant genes such as SLC7A11 and promoted the synthesis of GSH (Shih et al., 2003). Consequently, SLC7A11 is one of the most important transcriptional targets that can mediate the anti-oxidant response.

Transcription factor ATF4 regulates the expression of genes involved in amino acid metabolism, redox homeostasis and endoplasmic reticulum stress response (Pakos-Zebrucka et al., 2016; Sazonova et al., 2021). Translation of ATF4 mRNA is silenced by two short UORFs located in the 5′ untranslated region (UTR). The kinase that is catalyzed by eIF2α phosphorylation is activated by various cellular stresses, such as amino acid deprivation, endoplasmic reticulum stress, and viral infection (Koppula et al., 2018; Scalise et al., 2020). Inhibition of eIF2alpha phosphorylation levels led to inhibition of ATF4 mRNA translation and decreased ATF4 protein levels, while increased eIF2alpha phosphorylation levels led to enhanced ATF4 mRNA translation and increased ATF4 protein (Pathak et al., 2019). One upstream kinase of eIF2α is general control non-repressor-2 (GCN2), which is activated by free tRNAs in the presence of amino acid deprivation (Scalise et al., 2020). Thus, during amino acid deletion, GCN2 phosphorylates eIF2α, leading to the inhibition of protein synthesis in general, while increasing the translation of the specific transcription factor ATF4 (Ferraz-Bannitz et al., 2021). ATF4 associates with amino acid response elements (AARE) and promotes the transcription of genes related to amino acid metabolism and stress response, in particular SLC7A11, thereby enabling cells to cope with amino acid-limited conditions (Koppula et al., 2021b). Indeed, SLC7A11 expression can be strongly induced by deprivation of a variety of amino acids, and SLC7A11 expression induced by amino acid deprivation is mainly mediated by ATF4 (Koppula et al., 2021b). In summary, these data support that amino acid deletion induces SLC7A11 expression through the GCN2-eIF2α-ATF4 signaling axis.

Several studies have shown that the above transcription factors regulate downstream biological effects, including ferroptosis, antioxidant, and nutrient-dependent, through the regulation of SLC7A11 expression. SLC7A11 inhibits ferroptosis by increasing intracellular cystine and promoting glutathione synthesis (Dixon et al., 2012). By increasing SLC7A11 expression, ATF4 and NRF2 at least partially inhibit ferroptosis, whereas p53 stimulates ferroptosis by repressing SLC7A11 expression (Jiang et al., 2015; Fan et al., 2017; Roh et al., 2017). A study showed that p53 inhibits cystine uptake and leads to ferroptosis by suppressing SLC7A11, a component of the cystine/glutamate countertransport protein. In addition, mutant p533KR is defective in p53-dependent cell cycle arrest, apoptosis and senescence, but retains the ability to inhibit SLC7A11 expression, thereby regulating cystine metabolism and ferroptosis (Jiang et al., 2015).

#### 3.2.2. Lipid metabolism

Fatty acid metabolism is divided into anabolic and catabolic pathways, both of which are regulated by a variety of enzymes (Wakil and Abu-Elheiga, 2009). Fatty acid β-oxidation (FAO) in mitochondria normally consumes most of the fatty acids, leading to a reduction in lipid peroxidation. Cytoplasmic lipid droplets form the energy hub of almost all eukaryotic cells and when energy is available, they store energy in the form of esterified fatty acids and release them to local or distant tissues for oxidation (Goodman, 2019).

**Figure 4:**
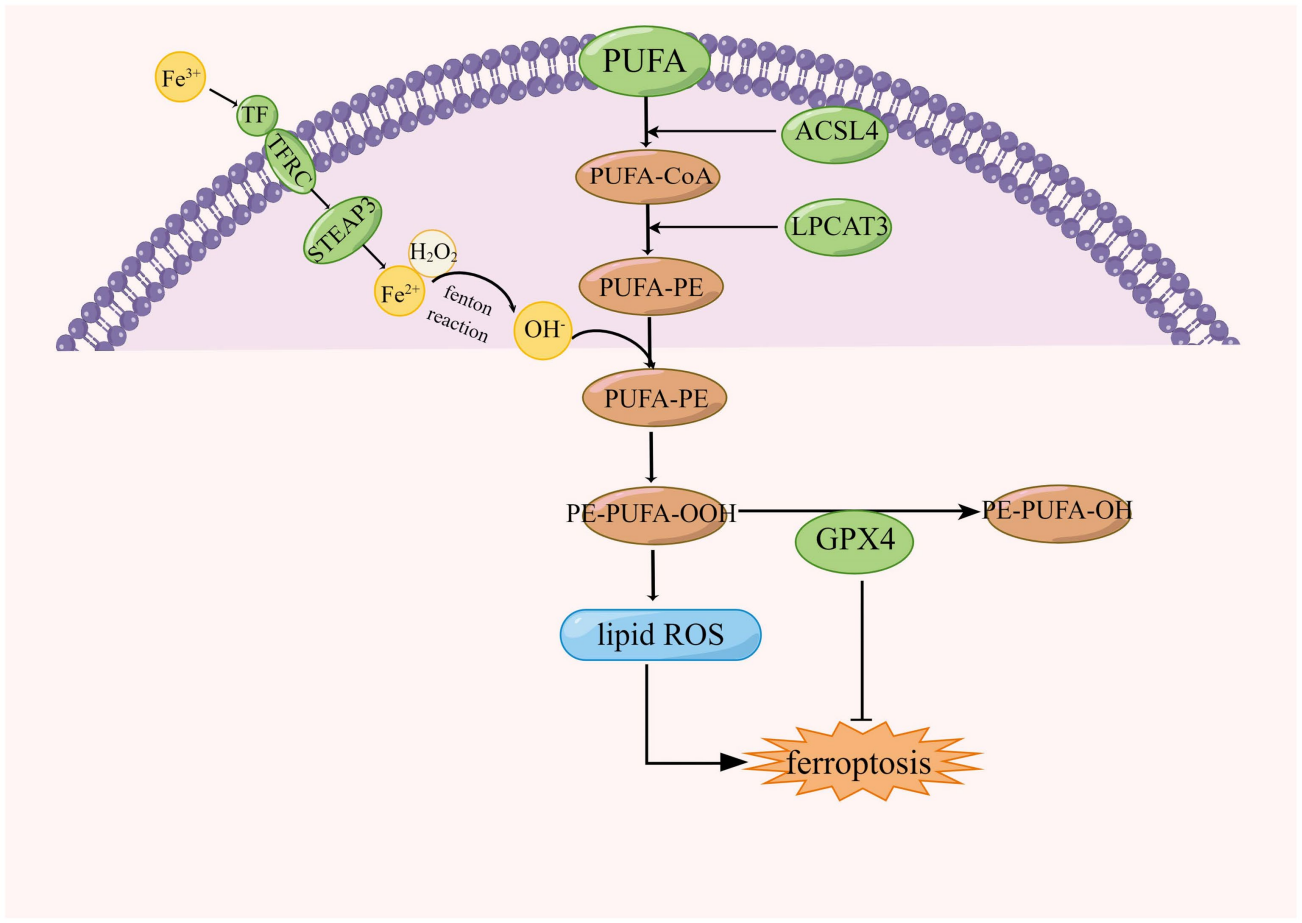
Regulation of lipid peroxidation in ferroptosis, ACSL4, acyl-coenzyme A synthase long chain family member 4; LPCAT3, lysophosphatidylcholine acyltransferase 3; CoA, coenzyme A; GPX4, glutathione peroxidase 4; and PUFA, polyunsaturated fatty acid.

Lipids play an important role in cellular functions, including membrane formation, energy production, intra- and intercellular signaling, and the regulation of cell death. Oxidation of phospholipids contributes to ferroptosis in cells (Yang et al., 2016). Lipid peroxides are produced in cells by three main pathways: first, lipid ROS from iron *via* a non-enzymatic Fenton reaction, second, lipid peroxides from oxidation and esterification of PUFAs, and third, lipid peroxides from iron-catalyzed lipid autoxidation. AA is a PUFAs that can be converted to adrenal acid (AdA) by prolonged enzymes. The accumulation of oxygenated AA-PE and AdA-PE evokes intracellular ferroptosis. Free PUFAs can be ultimately converted to phosphatidylethanolamine (PE)-PUFAs-OOH by three important enzymes, ACSL4, lysophosphatidylcholine acyltransferase 3 (LPCAT3), and lipoxygenases (LOXs; Jiang et al., 2020). The formation of lipid peroxides involves the formation of AA-PE from phosphatidylethanolamine (PE), an essential component of cell membranes, and arachidonic acid (AA), a PUFA, catalyzed by ACSL4 and LPCAT3, which is then peroxidized by iron-dependent LOX to form AA- OH -PE, the major actuator of ferroptosis (Protchenko et al., 2021; Liu et al., 2022), this is shown in Figure 4. ACSL family made up of proteins on the endoplasmic reticulum and outer membrane, ACSLs are responsible for the formation of fatty acid acyl coenzyme a esters from free long-chain fatty acids. The ACSL family contains five enzymes, ACSL4 is one of a family of five isomers, but only ACSL4 has a specific effect on ferroptosis (Yan and Zhang, 2019; Capelletti et al., 2020).

## 4. Crosstalk between excitotoxicity and ferroptosis

**Figure 5:**
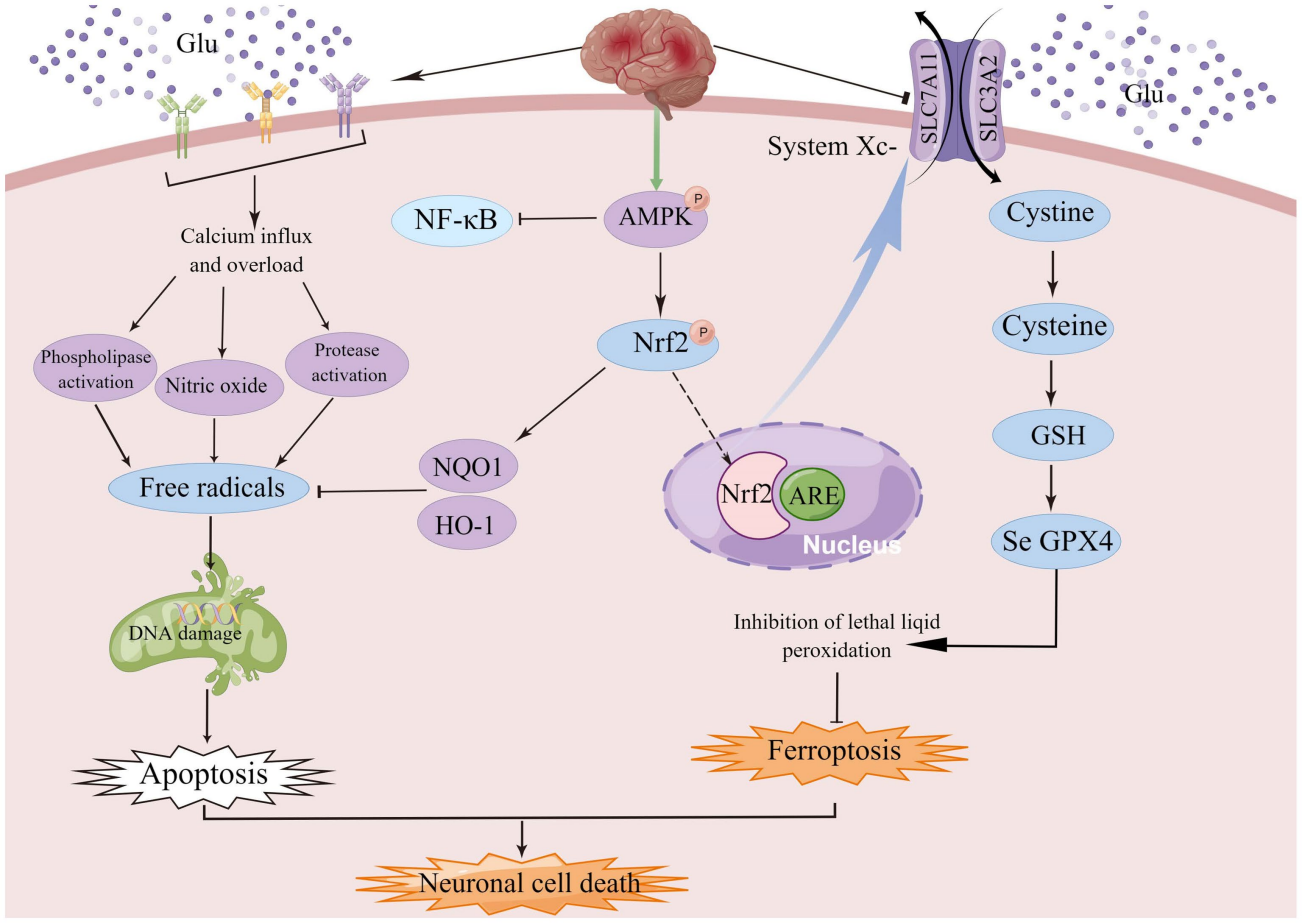
AMPK, a member of the serine/threonine kinase family, is an important endogenous defense factor against ischemia, and the Nrf2/ARE signaling pathway counteracts ischemia–reperfusion injury by inducing endogenous antioxidant defense factors and attenuating ROS production during reperfusion injury.

Excitotoxicity is mainly due to excessive glutamate release during ischemia leading to excessive activation of NMDAR, which leads to intracellular calcium overload, ROS-induced oxidative stress, mitochondrial dysfunction, and impaired membrane permeability (Yoo et al., 2017), Ca^2+^ overload *via* glutamate receptor-induced cPLA2 activation produces neurotoxic metabolites such as prostaglandins, leukotrienes, ROS, and platelet-activating factor *via* AA and lysophospholipid metabolism. It is known to be particularly sensitive to ferroptosis as AA and ADA are the main substrates of lipid peroxidation (Liu Y. et al., 2020; She et al., 2020; Hong et al., 2022), this is shown in Figure 5. The ROS generation cascade also includes the reaction of superoxide with nitric oxide to form peroxynitrite, hydrogen peroxide catalyzed by peroxidase to form hypochlorous acid, and the Fenton reaction catalyzed by iron to form hydroxyl radicals (Lin et al., 2016; Griendling et al., 2021). Mitochondrial ROS are essential not only for apoptosis but also for ferroptosis, although the common mechanisms determining the relationship between the two different types of cell death remain obscure (Gao et al., 2019; Lee et al., 2020; Li et al., 2021; Tang Z et al., 2021). However, there appears to be crosstalk between oxidative stress and ferroptosis during the development of ischaemic stroke. Excess glutamate accumulates extracellularly during stroke, causing excessive NMDAR activation and neuroexcitotoxicity, as well as inducing NMDAR-mediated iron uptake (Cheah et al., 2006), as BBB dysfunction during stroke allows iron-containing substances to enter the brain and accumulate in areas of ischaemic brain tissue prior to neurodegeneration (Helal, 2008; DeGregorio-Rocasolano et al., 2019). Thus, crosstalk between iron and glutamate in neurons is a target for intervention that cannot be ignored.

AMPK, a family member of serine/threonine kinases, is an invaluable endogenous defense factor against cerebral ischemia. During cerebral ischemia or hypoxia, the deprivation of energy and the consequent increase in the AMP/ATP ratio facilitates AMPK phosphorylation and initiates autophagy to bolster energy production (Jiang et al., 2014; Fang et al., 2018; Qin et al., 2022). AMPK activator A-769662 mimics the effects of silymarin and inhibits ROS production and neuronal cell death after OGD/R. In conclusion, these results suggest that silymarin-mediated neuroprotection may in part require activation of AMPK signaling (Xie et al., 2014). Under oxidative stress, Nrf2 is released from Keap1 and translocated to the nucleus, where it binds to the antioxidant response element (ARE) and upregulates the expression of NQO1 and HO-1 (Meng et al., 2014). The Nrf2/ARE signaling pathway counteracts ischemia–reperfusion injury by enhancing endogenous antioxidant defense factors and suppressing ROS production during reperfusion, which indicates that enhanced antioxidant properties can protect neurons (Young Park et al., 2019), and more importantly, NRF2 plays a key role in mediating iron/hemoglobin metabolism. NRF2 regulates the light and heavy chains of the iron storage protein ferritin (FTL/FTH1), and the iron transporter (SLC40A1) responsible for iron efflux from cells (Harada et al., 2011; Agyeman et al., 2012; Kerins and Ooi, 2018). NRF2 controls many of the enzymes that participate in glutathione synthesis and metabolism, including the catalytic and regulatory subunits of glutamate-cysteine ligase (GCLC/GCLM), glutathione synthase (GSS) and the subunit of the cystine/glutamate transporter xCT (SLC7A11), all of which are required for glutathione synthesis (Kwak et al., 2002; Sasaki et al., 2002). Among the multiple AMPK-related signaling pathways, the Nrf2 signaling pathway plays an important role in the regulation of genes and proteins with cytoprotective functions (Jiang et al., 2021). AMPK/NRF2 not only protects cells from oxidative stress damage, but also effectively regulates the expression of related genes to inhibit ferroptosis.

## 5. Treatment of ischemic stroke

### 5.1. Maintaining glutamate homeostasis

Glutamate is an important transmitter that plays a vital role in a variety of biological processes. Excess glutamate leads to over-stimulation of postsynaptic glutamatergic receptors, particularly NMDARs and AMPARs, allowing calcium to enter the cell, causing neuronal depolarisation and further neuronal death (Glotfelty et al., 2019), Inhibition of glutamate release, enhancement of glutamate clearance and blockade of glutamate receptors may be major directions for future stroke research. Methionine sulfoximine was found to be effective in inhibiting glutamate synthesis in mice (Ghoddoussi et al., 2010)，Dextromethorphan can inhibit glutamate release by inhibiting presynaptic voltage-dependent calcium channels (VDCC; Lin et al., 2009). In terms of glutamate clearance, ceftriaxone effectively increases GLT expression in glial cells and enhances glutamate clearance (Lai et al., 2011). NMDAR inhibitors are widely studied drugs, and magnesium sulfate has shown a prominent role in protecting neurons from excitotoxicity by inhibiting NMDAR, reducing the transmission of the excitatory neurotransmitter glutamate, and reducing the inward flow of calcium ions (Ovbiagele et al., 2003). Memantine is a non-competitive NMDAR inhibitor. Memantine selectively blocks the over-activation of NMDAR in excitotoxicity and memantine increases the upregulation of brain-derived neurotrophic factor (BDNF) and glial cell-derived neurotrophic factor (Martínez-Coria et al., 2021). A meta-analysis showed no improvement in key outcome indicators and mortality in acute ischaemic stroke treated with magnesium sulfate (Avgerinos et al., 2019), it seems to be due to the fact that it is more difficult to treat effectively within the time window. Peritoneal dialysis has been demonstrated to decrease peripheral blood glutamate levels in rats with cerebral ischemia (Godino Mdel et al., 2013). Therefore, inhibition of glutamate synthesis, enhancement of glutamate clearance and inhibition of glutamate receptors play an important role in the protection of ischemic stroke.

### 5.2. Inhibition of calcium increase and oxidative stress

Calcium ions are a commonly present second messenger that regulates a variety of activities such as excitability, cytoplasmic division, motility, transcription and apoptosis in eukaryotic cells (Bootman, 2012; Carafoli and Krebs, 2016; Pchitskaya et al., 2018). The initial calcium influx following excitotoxic glutamate stimulation is known to trigger a secondary intracellular calcium overload, and this secondary response strongly correlates with neuronal death (Randall and Thayer, 1992; Tymianski et al., 1993). The plasma membrane sodium-calcium exchanger (NCX) is an essential modulator of intracellular calcium levels, using the force of sodium influx to expel calcium ions. The action of the NCX partially restores calcium ions to physiological levels following glutamate stimulation (White and Reynolds, 1995). Another major player in intracellular calcium homeostasis is the mitochondria, which can restore intracellular calcium concentrations by absorbing large amounts of calcium themselves (Valdinocci et al., 2019; Sanz-Morello et al., 2021), and by facilitating ATP-dependent calcium extrusion (Budd and Nicholls, 1996; White and Reynolds, 1996). Mitochondrial uptake of calcium in response to excitotoxic glutamate stimulation leads to ROS production (Castilho et al., 1999)，excessive opening of the mitochondrial membrane permeability transition pore leads to a decrease in mitochondrial membrane potential (Castilho et al., 1999), induction of neuronal death (Stout et al., 1998; Ward et al., 2000). Therefore, inhibition of calcium increase and oxidative stress may be a therapeutic target in ischaemic stroke. Studies have demonstrated that the influx of calcium ions into cells during excitotoxicity is an essential pathway causing cell death, so interruption of the inward flow of extracellular calcium ions and decreasing the degree of calcium overload could theoretically protect neuronal cells to a large extent. Calcium antagonists have been proven in animal experiments to dramatically reduce the size of brain infarcts in rats and to have a protective neuronal effect (Zapater et al., 1997; Choi et al., 2011). Results of a meta-analysis show no effect of calcium antagonists on primary patient outcomes or death, and researchers show no evidence to support the use of calcium antagonists in patients with ischaemic stroke as beneficial (Zhang et al., 2019). 4,1-benzothiazoles are non-calcium antagonist drugs that reduce calcium levels in neurons by modulating mitochondria (Viejo et al., 2021). Calcium antagonists continue to be the subject of stroke research, although they have not achieved the desired results in clinical trials, probably because of intolerable side effects, low efficacy and short treatment windows. Uric acid is the final oxidation product of purine catabolism in the body and accounts for about two-thirds of the total antioxidant capacity of plasma (Becker, 1993). Uric acid has been shown to prevent glutamate-induced cell death *in vitro* and to inhibit ROS and RNS to reduce infarct size and improve prognosis in rodents after transient or permanent cerebral ischemia (Squadrito et al., 2000; Romanos et al., 2007; Onetti et al., 2015; Chamorro et al., 2016). Edaravone is an antioxidant drug that has been shown to scavenge the accumulation of free radicals and lipid peroxidation products in both clinical trials and basic experiments (Kasuya et al., 2014; Fidalgo et al., 2022).

### 5.3. Inhibition of ferroptosis

**Figure 6:**
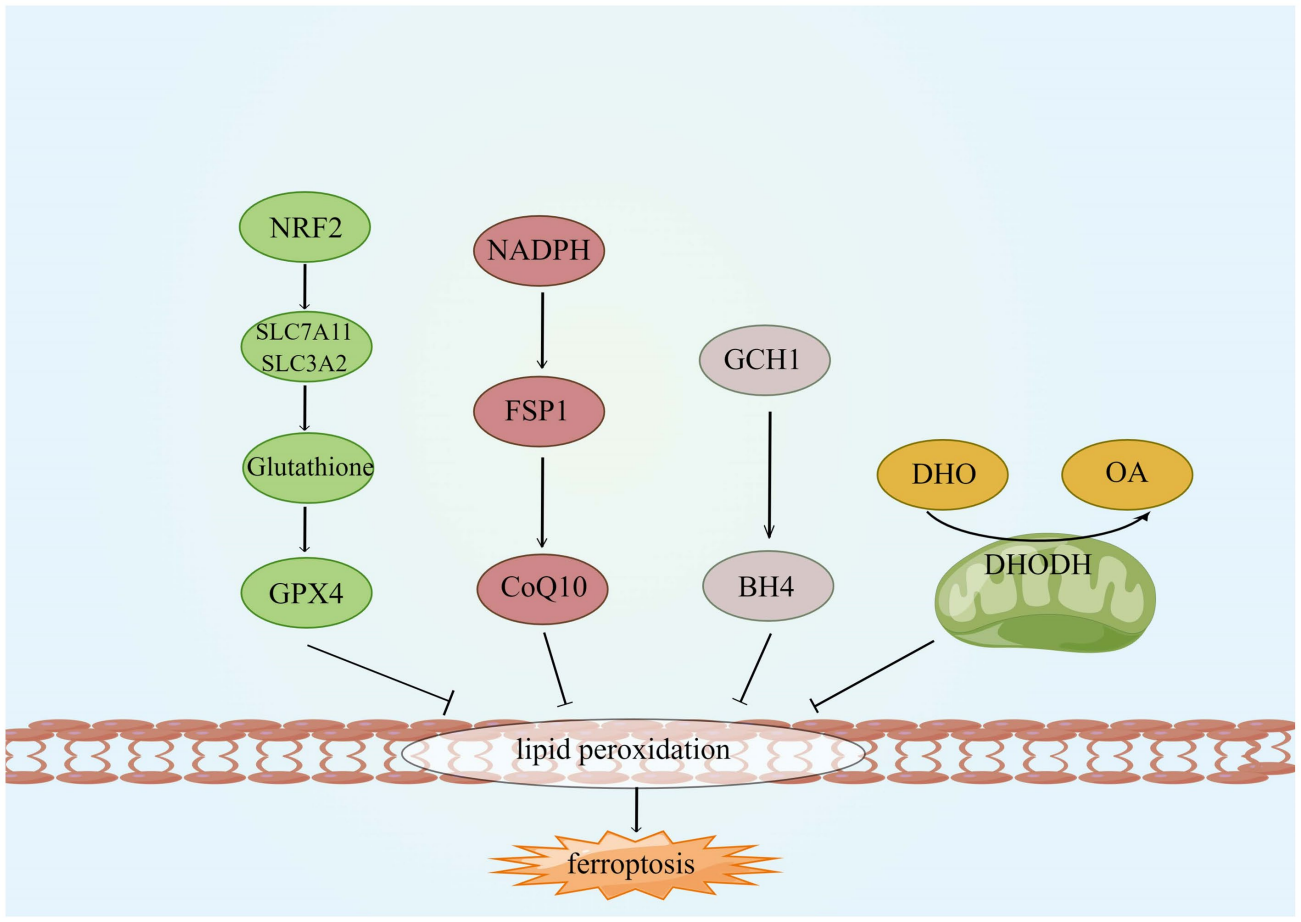
Four ferroptosis defense pathways. GCH1, GTP cyclohydrolyse-1; FSP1, ferroptosis suppressor protein 1; CoQ10, coenzyme Q; DHODH, dihydroorotate dehydrogenase; DHO, dihydroorotate; OA, orotate; GSH, glutathione; and BH4, tetrahydrobiopterin.

As ferroptosis is characterized by excessive lipid peroxidation, iron chelators, lipophilic antioxidants, and lipid peroxidation inhibitors can inhibit ferroptosis (Wang K. et al., 2022). Four types of ferroptosis inhibitors have been identified: GPX4 specifically catalyzes the loss of lipid peroxide oxidation activity in a GSH-dependent manner, FSP1 converts ubiquitin ketone on cell membranes to reduced ubiquitin, which can inhibit peroxidation and prevent iron droopy, GCH1/BH4 pathway is an endogenous antioxidant pathway, GCH1 protects cells from ferroptosis mainly through the antioxidant effect of BH4, and DHODH protects cells from ferroptosis in mitochondria by regulating the production of dihydrobisquinone in the inner mitochondrial membrane (Wang D. et al., 2022), this is shown in Figure 6. Some compounds inhibit ferroptosis directly or indirectly by targeting lipid peroxidation and iron metabolism (Chen G. et al., 2020). Both iron chelators (2,2′-pyridine, deferoxamine, deferoxamine mesylate) and inhibitors of lipid peroxides (Ferrostatin-1, Liproxstatin-1, Vitamin E) suppressed ferroptosis (Čepelak et al., 2020; Li et al., 2020). In additional, GSH, GPX4, heat shock protein β-1 and Nrf2 negatively modulate ferroptosis by restraining ROS production and repressing cellular uptake of iron (Sun et al., 2015; Qin et al., 2021). DFO, the most widely used iron chelator approved by the FDA, inhibits lipid peroxide chelation by inhibiting the Fenton reaction, and one study found that DFO effectively protects neurons by increasing the expression of hypoxia-inducible factor 1 (HIF-1; Baranova et al., 2007; Zhang et al., 2021). The widely used RTAs are ferrostatin-1 and liproxstatin-1, which can inhibit lipid peroxidation linked to ferroptosis (Han et al., 2020). ACSL4 is a crucial enzyme for AA and ADA esterification and is most probably an essential target for the inhibition of ferroptosis. Thiazolidinediones (TZNs) have been shown to potentially inhibit the activity of ACSL4 specifically and to prevent ferroptosis (Doll et al., 2017; Zhang et al., 2021).

## 6. Conclusion and perspectives

Excessive accumulation of glutamate not only leads to excitotoxicity, but also to ferroptosis, Therefore, maintaining glutamate homeostasis is essential to inhibit excitotoxicity and ferroptosis. We can see a large number of articles using glutamate modeling to study the mechanism of excitotoxicity, most of which only look at the increase in calcium ions and mitochondrial dysfunction caused by excitotoxicity. In fact, the researchers used glutamate to create excitotoxic cell models that also caused ferroptosis, and excitotoxicity may be only part of the equation. Therefore it is also important to focus on neuronal ferroptosis when using glutamate for modeling in future studies. Neuronal excitotoxicity or ferroptosis can be effectively inhibited by compounds in most basic studies, particularly glutamate receptor inhibitors, but the role in clinical trials has been greatly reduced, probably mainly due to the failure to treat effectively within the time window in clinical trials. A number of influencing factors are essential, including informed patient consent, family cooperation and a well-established hospital system of care may all be influential in clinical trials. We remain confident in developing natural compounds that regulate both ferroptosis and excitotoxicity in future basic practice and further investigating their complex mechanisms and regulatory effects.

## Author contributions

GF and YH conceived and designed this review. ML and JL contributed to the collection of literature and related information. GF and ML wrote the manuscript and examined the charts and the grammar of the manuscript. JL and YH provide guidance throughout the manuscript preparation process. All authors contributed to the article and approved the submitted version.

## Funding

This project is supported by the Young Qihuang scholars program of National Administration of Traditional Chinese Medicine.

## Conflict of interest

The authors declare that the research was conducted in the absence of any commercial or financial relationships that could be construed as a potential conflict of interest.

## Publisher’s note

All claims expressed in this article are solely those of the authors and do not necessarily represent those of their affiliated organizations, or those of the publisher, the editors and the reviewers. Any product that may be evaluated in this article, or claim that may be made by its manufacturer, is not guaranteed or endorsed by the publisher.
